# Supplementary material for: Enhanced Energy Storage Performance of PVDF-Based Composites Using BN@PDA Sheets and Titania Nanosheets
Source: Materials (Basel). 2022 Jun 21;15(13):4370. doi: 10.3390/ma15134370 (PMC9267653; doi:10.3390/ma15134370)
Supplement: Supplementary file 1 [file materials-15-04370-s001.zip › materials-1737093-supplementary.pdf]

Supplementary Material

Enhanced energy storage performance of PVDF based composites using BN@PDA sheets and titania nanosheets

Congcong Zhu[1, 2], Jinghua Yin[1, 2, *], Yu Feng[2], Jialong Li[3], Yanpeng Li[1], He Zhao[2], Dong Yue[1] and Xiaoxu Liu[3, **]

[1] School of Materials Science and Chemical Engineering, Harbin University of Science and Technology, Harbin 150080, P. R. China.

[2] Key Laboratory of Engineering Dielectrics and Its Application, Ministry of Education, Harbin University of Science and Technology, Harbin 150080, P. R. China.

[3] School of Materials Science and Engineering, Shaanxi University of Science and Technology, Xi'an, 710021, China.

Correspondence to：*E-mail: yinjinghua1@126.com, **E-mail: xiaoxuliu@sust.edu.cn

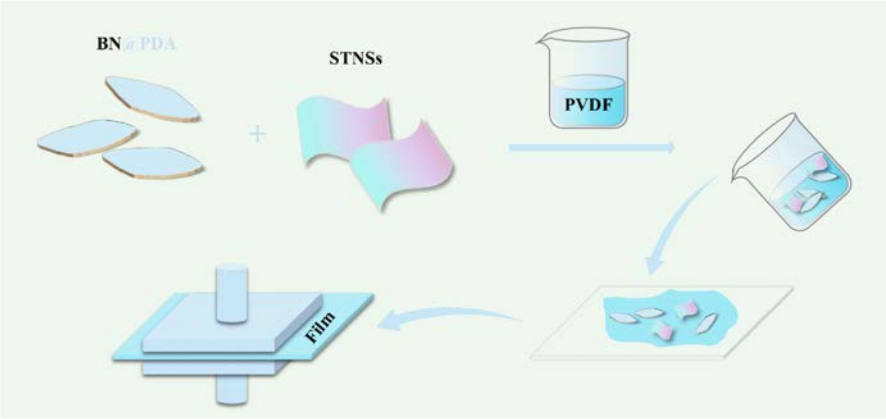

Figure S1. Schematic illustration of synthesizing the PVDF/BN@PDA−STNSs composites.

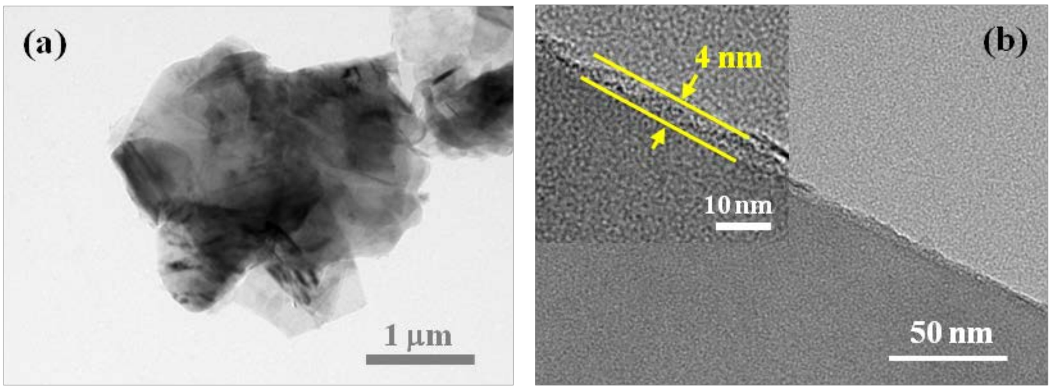

Figure S2. (a) Low power TEM image and (b) high power TEM image of 2D BN@PDA sheets.

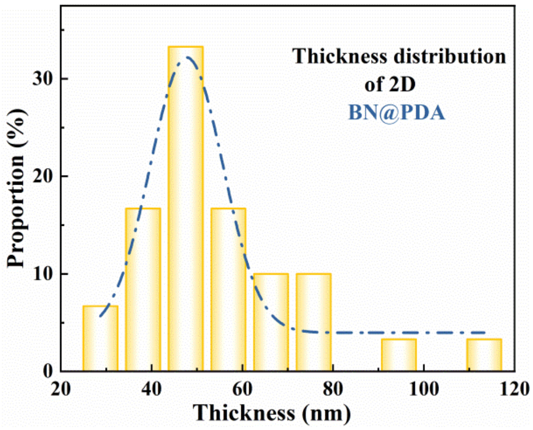

Figure S3. The thickness distribution of 2D BN@PDA sheets.

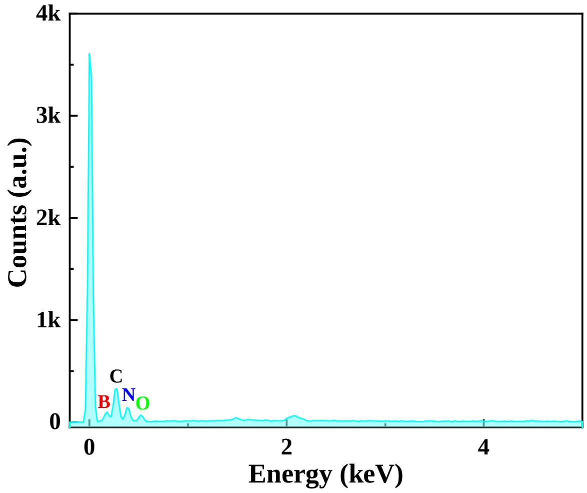

Figure S4. The EDS spectrum of BN@PDA.

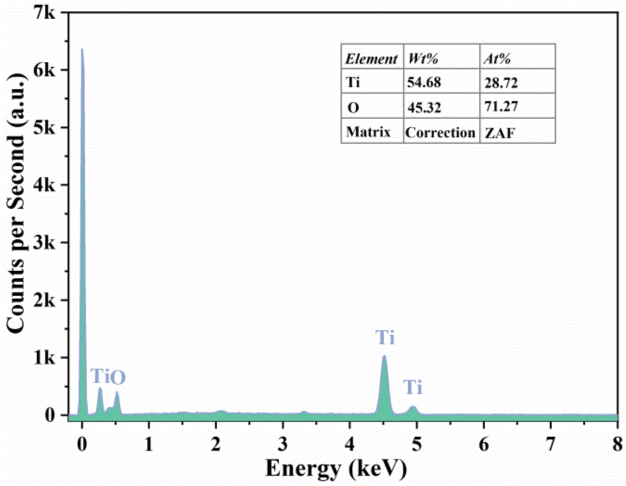

Figure S5. The EDS spectrum of STNSs.

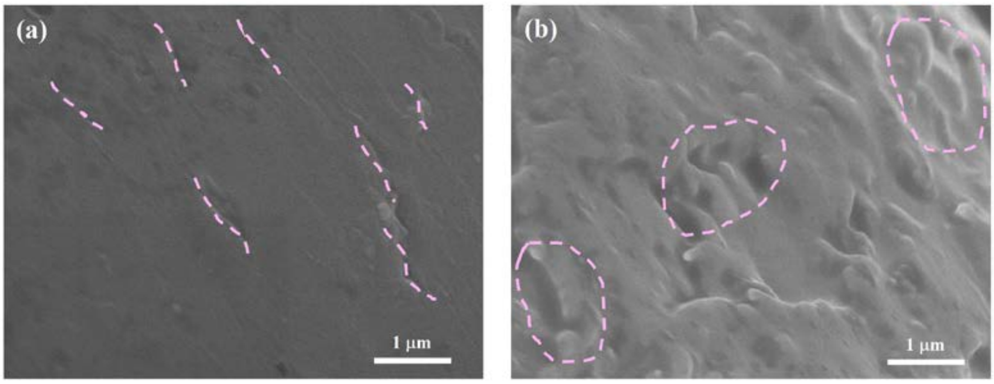

Figure S6. Cross-section morphology of the (a) 3 wt% and (b) 10 wt% PVDF/BN@PDA composite.

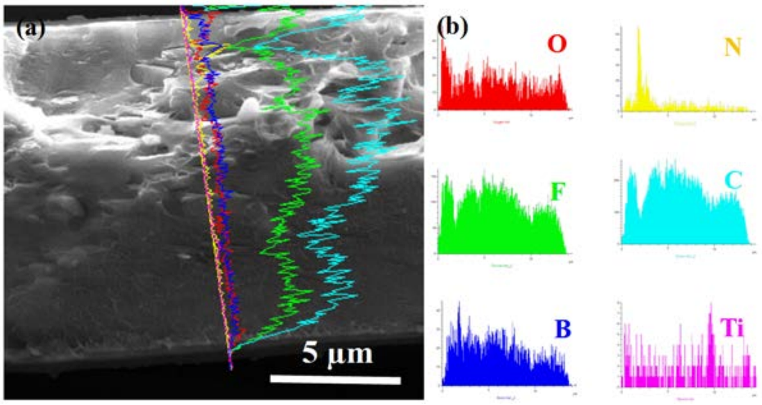

Figure S7. (a) Cross-section morphology and (b) mapping of 3 wt% PVDF/BN−STNSs composite.

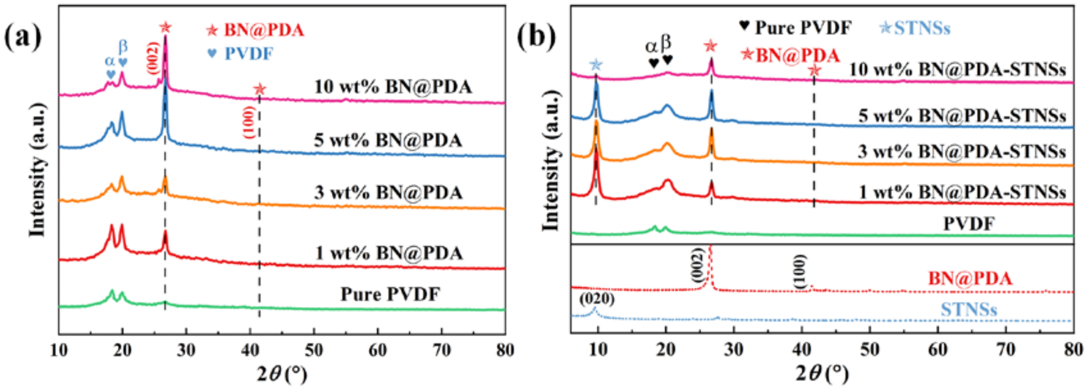

Figure S8. The XRD patterns of (a) PVDF/BN@PDA and (b) PVDF/BN@PDA−STNSs composites.

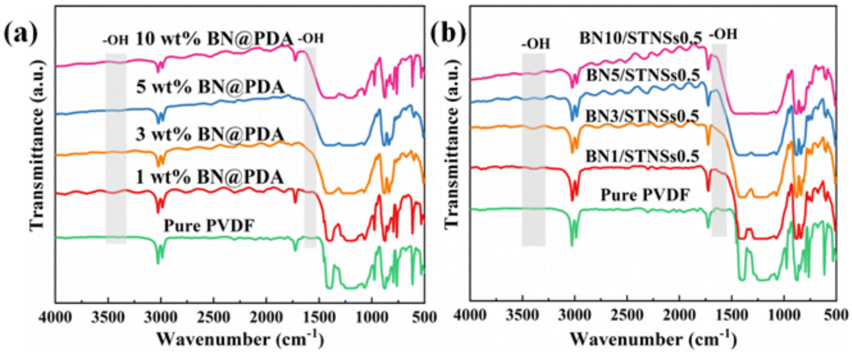

Figure S9. FTIR spectra of (a) PVDF/BN@PDA and (b) PVDF/BN@PDA−STNSs composites.

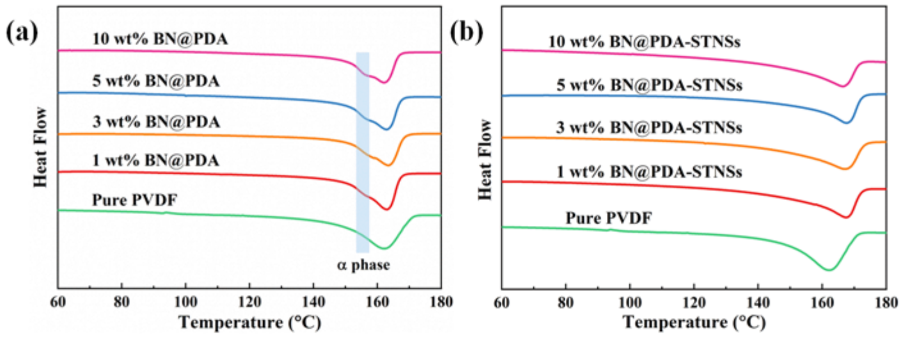

Figure S10. The DSC endothermic curves of (a) PVDF/BN@PDA and (b) PVDF/BN@PDA−STNSs composites.

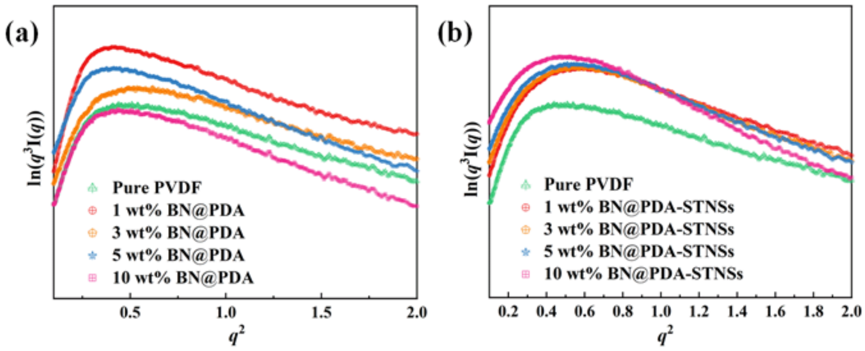

Figure S11. Porod curves of (a) PVDF/BN@PDA and (b) PVDF/BN@PDA−STNSs composites.

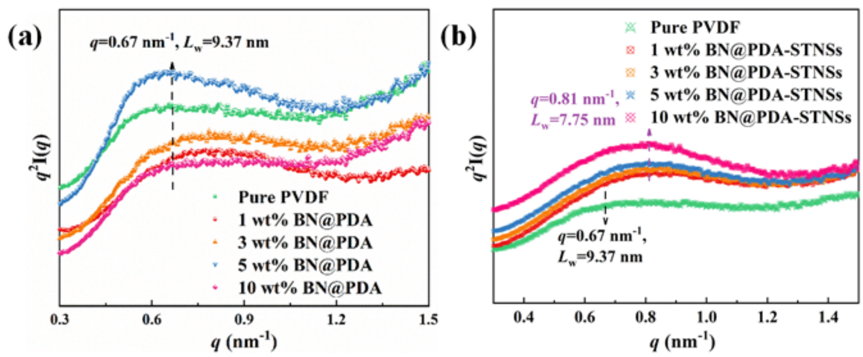

Figure S12. Lorentz corrected SAXS profiles of (a) PVDF/BN@PDA and (b) PVDF/BN@PDA−STNSs composites.

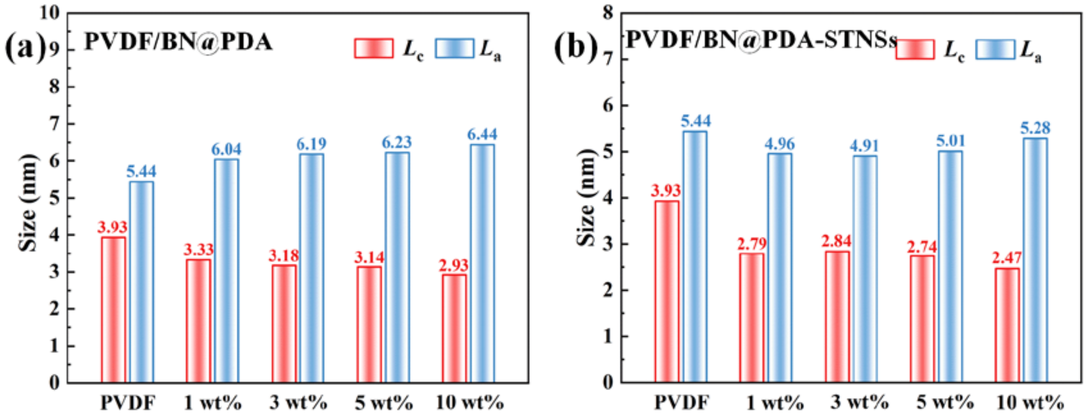

Figure S13. The L_c and L_a of (a) PVDF/BN@PDA and (b) PVDF/BN@PDA−STNSs composites.

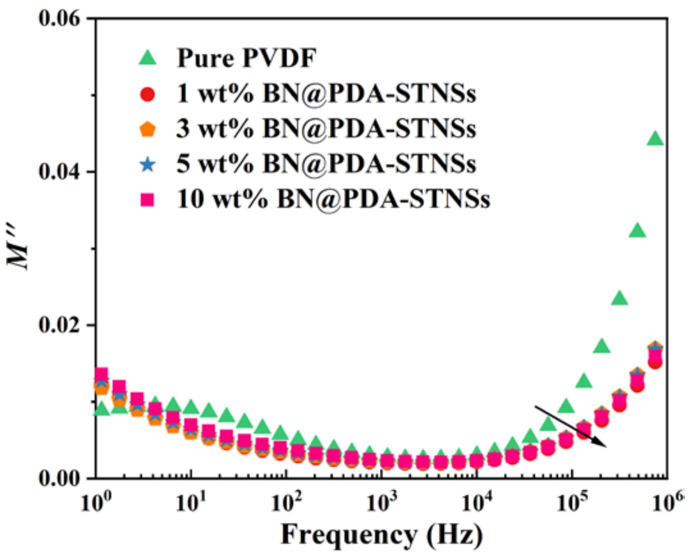

Figure S14. *M''* of the PVDF/BN@PDA−STNSs ternary composites.

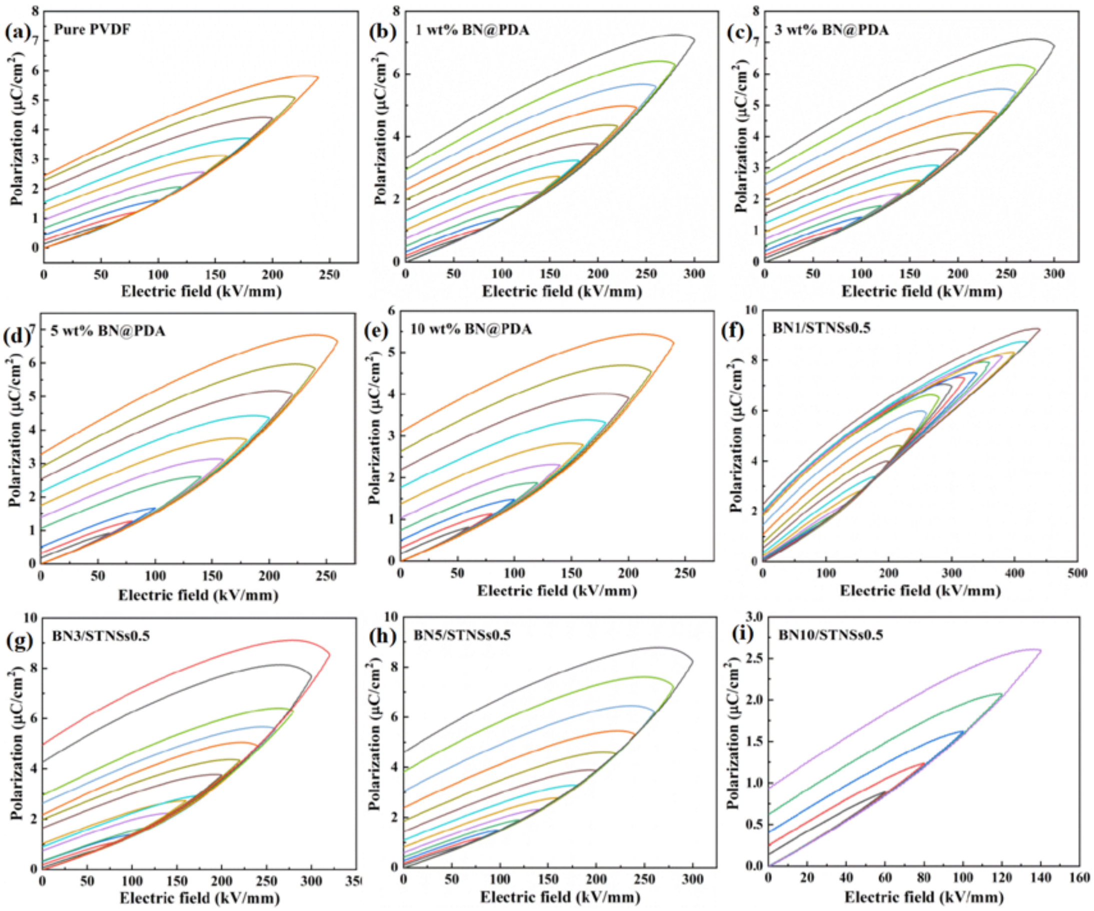

Figure S15. Hysteresis loops of pure PVDF, PVDF/BN@PDA and PVDF/BN@PDA−STNSs composites.

Table S1 DSC results and $F(\beta)$ of the composites.

Samples	Contents	T_m (°C)	ΔH_m (J/g)	X_c (%)	$F(\beta)$
	0 wt%	161.9	43.7	41.8	52.3
	1 wt%	162.6	35.3	35.6	59.9
PVDF/BN@PDA	3 wt%	163.1	35.0	34.0	60.5
	5 wt%	162.6	34.8	33.5	60.3
	10 wt%	161.8	28.9	31.3	53.4
	1 wt%	167.2	37.5	35.9	60.5
PVDF/BN@PDA-STNSs	3 wt%	167.3	38.2	36.6	60.7
	5 wt%	167.5	37.0	35.4	60.3
	10 wt%	166.1	33.2	31.8	59.4
